# Human–AI emotional resonance in generative music: a mini-review of authorship attribution, affective response, and aesthetic evaluation

**DOI:** 10.3389/fpsyg.2026.1889696

**Published:** 2026-08-26

**Authors:** Wenting He, Yuyi Sun

**Affiliations:** 1 Zhengzhou University, Zhengzhou, China; 2 Ludong University, Yantai, China

**Keywords:** aesthetic evaluation, AI-generated music, authorship attribution, emotional resonance, expectancy effect, generative AI, human–AI interaction, music psychology

## Abstract

Generative artificial intelligence is increasingly involved in music creation, raising important questions about how listeners emotionally respond to music that is generated by, or attributed to, AI. This mini-review synthesizes recent empirical research on listeners' emotional, aesthetic, and physiological responses to AI-generated music. Existing evidence suggests that AI systems can reproduce some acoustic cues associated with basic emotional categories and may elicit measurable affective or attentional responses under specific conditions. However, listeners' evaluations are shaped not only by acoustic features but also by authorship attribution. Several studies report lower liking, perceived quality, authenticity, or engagement when music is attributed to AI, although null and occasionally reversed label effects have also been observed. To explain this pattern, this review proposes a dual-process framework that distinguishes between low-level acoustic–emotional processing and high-level source-based appraisal involving agency, intentionality, authenticity, and human experience. Overall, current evidence supports a partial dissociation between immediate affective responses and higher-order cognitive evaluation. Whether AI-generated music can support deeper forms of emotional resonance, such as long-term attachment, perceived understanding, personal meaning, or source-based empathy, remains insufficiently established. Future research should use identical-audio attribution designs, stricter audio-quality matching, cross-genre comparisons, and longitudinal listening paradigms.

## 1 Introduction

Generative artificial intelligence is changing the way people create, produce and listen to music. Recent text-to-music systems can generate stylistically coherent musical excerpts from natural-language prompts. This development translates a fundamental question—whether emotionally meaningful music requires a human creator with embodied experience—into a testable psychological problem. However, emotional responses to music are not determined solely by acoustic parameters; they also involve expectations, memory, cognitive appraisal, physiological reactions, and inferences about the creator's intention and agency (Huron, 2006; Juslin and Västfjäll, 2008; Meyer, 1956; Thompson et al., 2023).

This forms a core paradox in the music generated by artificial intelligence. If the algorithm can reproduce acoustic cues associated with happiness, sadness, or tension, will the audience experience the same emotional resonance as when enjoying human music? Existing research suggests that perceived authorship can influence aesthetic and emotional evaluation under some experimental conditions. Several studies report lower liking, engagement, emotional valence, or perceived quality when music is attributed to AI, although these effects are not uniform across genres, outcomes, or experimental designs (Ansani et al., 2025; Chia et al., 2025; Shank et al., 2023). Physiological evidence is also mixed. AI-origin beliefs have been associated with threat-related interpretations and changes in autonomic activity, whereas actual AI-generated soundtracks have sometimes elicited greater pupil dilation or arousal in audiovisual contexts (Dunham et al., 2025; Fišer et al., 2025). These findings arise from different designs and should not be treated as directly equivalent.

Therefore, this review synthesizes recent empirical evidence concerning listeners' liking, perceived quality, authenticity, creativity, emotional valence, arousal, emotional intensity, engagement, and physiological or attentional responses to AI-generated or AI-attributed music. It further examines whether immediate acoustic–affective responses can be distinguished from higher-order evaluations involving perceived agency, intentionality, authenticity, human experience, and creative identity.

## 2 Review methodology

### 2.1 Review design and scope

This mini-review adopted a structured narrative review approach with a transparent search, eligibility-assessment, and evidence-synthesis procedure. Relevant reporting items from the PRISMA 2020 statement were used to improve the transparency of study identification, screening, and inclusion (Page et al., 2021). However, the review was not registered and was not designed as a comprehensive systematic review or meta-analysis. Its purpose was to synthesize recent empirical evidence concerning listeners' affective, aesthetic, attentional, and physiological responses to music that was generated by, performed by, co-created with, or attributed to artificial intelligence.

The review focused on two related but analytically distinct bodies of evidence. The first concerned authorship attribution and source-label effects, including whether AI-related source information influenced liking, perceived quality, creativity, authenticity, expressiveness, engagement, or meaning-making. The second concerned the capacity of AI-generated music to convey or induce emotional responses, including perceived emotion, felt valence, arousal, emotional intensity, attentional responses, and physiological activity.

### 2.2 Search strategy

The search covered publications from 1 January 2019 to 21 July 2026. The following sources were consulted: Web of Science Core Collection, Scopus, APA PsycINFO, PubMed, ACM Digital Library, and IEEE Xplore. Supplementary searches were conducted through publisher websites, DOI records, backward reference searching, and forward citation searching.

Two complementary search pathways were used. The first targeted studies of authorship attribution and aesthetic evaluation:

(“AI-generated music” OR “AI generated music” OR “AI-composed music” OR “AI composed music” OR “AI-performed music” OR “AI performed music” OR “computer-generated music” OR “machine-generated music” OR “algorithmically generated music” OR “human–AI co-created music”) AND (listener^*^ OR audience^*^ OR participant^*^ OR “human evaluation” OR “user study” OR “listening experiment”) AND (authorship OR attribution OR “source label” OR “origin belief” OR liking OR preference^*^ OR quality OR aesthetic^*^ OR authenticity OR creativity OR expressiveness OR engagement OR empathy OR meaning).

The second pathway targeted emotional conveyance, emotional induction, and physiological response:

(“AI-generated music” OR “AI generated music” OR “AI-composed music” OR “AI composed music” OR “AI-performed music” OR “computer-generated music” OR “machine-generated music” OR “algorithmically generated music” OR “emotion-conditioned music”) AND (listener^*^ OR audience^*^ OR participant^*^ OR “human evaluation” OR “listening experiment”) AND (emotion^*^ OR affect^*^ OR “emotion recognition” OR “emotion perception” OR “emotion conveyance” OR “emotion induction” OR valence OR arousal OR “emotional intensity” OR physiological OR autonomic OR pupil^*^ OR pupillometry OR “heart rate” OR electrodermal).

Searches were restricted to publications dated between 2019 and 2026 and to peer-reviewed journal articles or full conference papers where database filters were available.

An initial pilot search using broad combinations of the terms “artificial intelligence,” “music,” “performance,” “participant,” and “response” retrieved a large number of irrelevant records, including studies of algorithm performance, music recommendation, classification, and non-musical applications. These pilot-search results were not included in the final study-selection counts. The final candidate corpus was developed using the refined search pathways described above and was supplemented through reference and citation checking.

### 2.3 Eligibility criteria and study selection

Studies were included when they met all of the following criteria: (a) the musical material was generated, composed, performed, assisted, or co-created by AI, or the music was experimentally attributed to an AI or human source; (b) the study included human listeners or participants; (c) at least one listener-level outcome was reported, including liking, preference, quality, creativity, authenticity, expressiveness, engagement, emotional valence, arousal, emotional intensity, emotion recognition, emotion induction, meaning-making, attentional response, or physiological response; (d) the report was published as a peer-reviewed journal article or a formal full conference paper; and (e) the publication date fell between 1 January 2019 and 21 July 2026.

Studies were excluded if they were purely theoretical or conceptual; evaluated only technical generation metrics; focused on recommendation, classification, tagging, retrieval, or copyright detection without listener-level outcomes; used speech, voice cloning, lyrics in isolation, or non-musical sounds as the principal stimulus; or were published only as preprints, working papers, dissertations, technical reports, abstracts, posters, or industry reports. Where both a preprint and a formally published version of the same study were identified, only the published version was retained.

The database searches identified 187 records: Web of Science Core Collection (*n* = 46), Scopus (*n* = 58), APA PsycINFO (*n* = 14), PubMed (*n* = 11), ACM Digital Library (*n* = 26), and IEEE Xplore (*n* = 32). Supplementary searching through publisher websites, DOI records, backward reference checking, and forward citation searching identified nine additional records. After 72 duplicate records were removed, 124 unique records remained for title and abstract screening. Ninety-eight records were excluded at this stage because they did not concern AI-related music, did not include human listeners, or did not report eligible listener-level outcomes. The remaining 26 reports were retrieved and assessed in full text. Eight reports were excluded: three were preprints or working papers, one was not a fully peer-reviewed publication, two had insufficient information to verify formal publication status, and two were technical studies or did not report eligible listener-level outcomes. The final evidence synthesis therefore included 18 peer-reviewed journal articles or formal full conference papers. Technical and conceptual sources cited for background or framework development were not counted among the 18 included listener studies.

**Figure 1:**
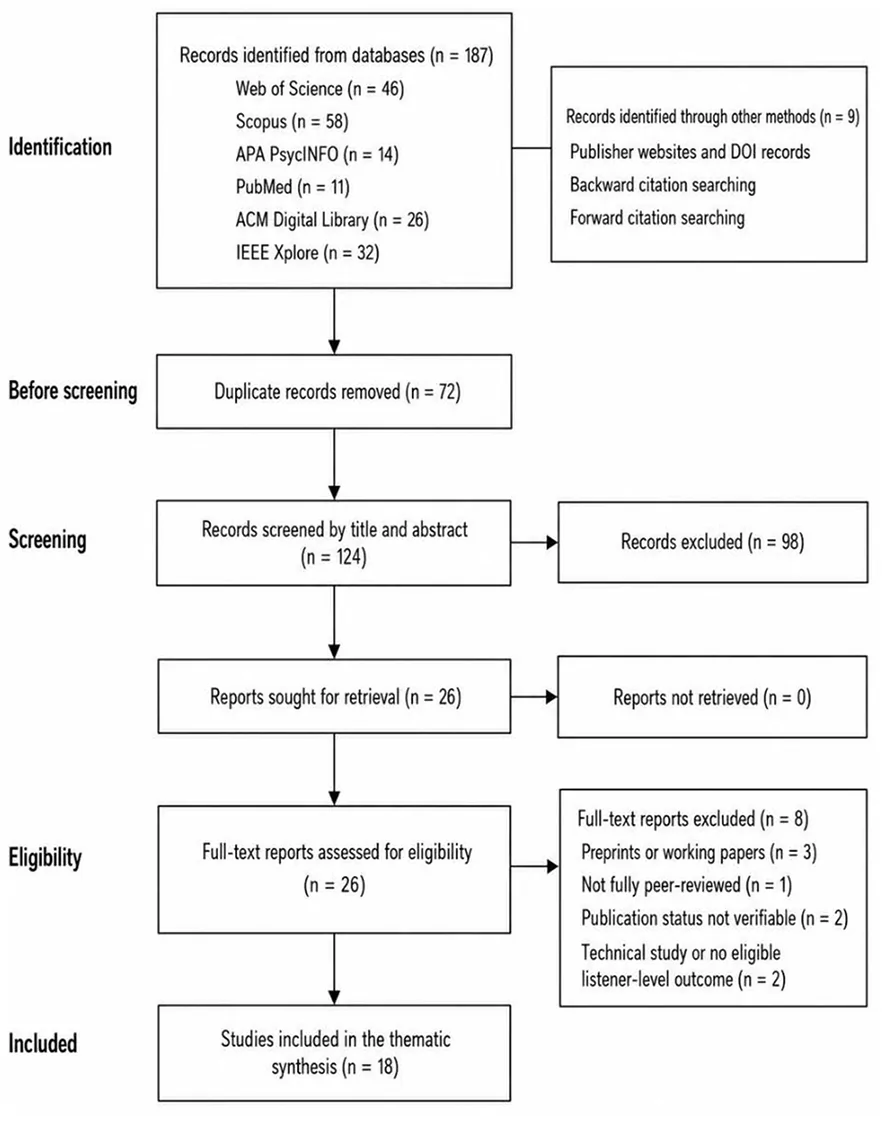
Study-selection procedure for the identification, screening, and inclusion of studies.

One author conducted the initial eligibility assessment and documented the reason for excluding each report. Records for which eligibility was uncertain were reviewed with a second author, and final decisions were reached through discussion and consensus. The study-selection procedure is summarized in Figure 1.

### 2.4 Data extraction and thematic synthesis

A structured data-extraction framework was used to ensure that the included studies were summarized consistently. For each report, the following information was extracted: author and publication year, publication type, sample size and participant characteristics, research design, musical genre, stimulus type, type of AI involvement, authorship or source-label manipulation, listener-response measures, principal findings, and reported methodological limitations. The extraction process also recorded whether a study used identical musical material with different AI or human source labels, compared different AI-generated and human-created musical excerpts, or evaluated multiple AI-generation systems without a human comparison condition.

Particular attention was given to the distinction between authorship-attribution designs and direct stimulus-comparison designs. Studies using identical audio with different source labels provide relatively strong evidence concerning source-label, expectancy, or authorship-attribution effects because the acoustic properties of the musical material are held constant. In contrast, studies comparing actual AI-generated and human-created music cannot isolate authorship attribution from differences in composition quality, performance quality, production value, model version, genre fit, familiarity, mixing, or timbral characteristics. Findings from these two types of design were therefore interpreted separately.

The included studies were heterogeneous in their research designs, musical genres, AI systems, stimulus duration, participant populations, and outcome measures. The reported outcomes included liking, perceived quality, creativity, authenticity, expressiveness, engagement, emotional valence, arousal, emotional intensity, emotion recognition, emotion induction, meaning-making, attentional responses, and physiological activity. Because of this methodological and conceptual heterogeneity, and because the available studies did not provide a sufficiently consistent set of effect-size estimates, a statistical meta-analysis was not considered appropriate.

The findings were instead synthesized thematically. Studies were organized into three principal evidence domains: (a) authorship attribution and source-label effects; (b) emotional conveyance, emotional induction, and physiological or attentional responses; and (c) individual, genre-related, cultural, and methodological boundary conditions. Within each domain, the synthesis compared areas of convergence and inconsistency, considered the strength of the underlying research designs, and distinguished findings directly supported by empirical evidence from broader theoretical interpretations.

The extracted study characteristics and principal findings are summarized in Table 1.

**Table 1:** Characteristics and principal findings of the empirical studies included in the review.

Study	Sample	Design and musical stimuli	AI involvement or authorship manipulation	Outcome measures	Principal findings	Main methodological limitation
Tigre Moura and Maw (2021)	Survey: N = 446; experiment: *N* = 86	Survey and 2 × 2 laboratory experiment using AI-composed songs	AI-composed music presented with human- vs. AI-composition information	Music perception, attitudes, credibility, purchase intention	General attitudes toward AI-composed music were relatively negative, particularly among music professionals; the experimental source narrative did not consistently alter music evaluations.	Small experimental sample and limited musical materials; broad attitudes and direct listening evaluations were combined.
Hong et al. (2021)	*N* = 299	2 × 2 × 2 online experiment using classical and electronic dance music	AI-composed music combined with expectancy confirmation or violation, positive or negative information, and genre	Evaluation of AI music, acceptance of creative AI, expectancy responses	Greater acceptance of AI creativity was associated with more favorable music evaluation; effects of expectancy information varied by genre and information valence.	The design examined expectations surrounding AI composition rather than a clean identical-audio AI-vs.-human label effect.
Hong et al. (2022)	*N* = 124	2 × 2 experiment using AI-composed songs	Manipulation of the AI composer's humanlike attributes and autonomous creative capacity	Acceptance of AI as a musician, song appreciation, attitudes and affective response	Humanlike attributes increased acceptance of AI as a musician; perceived musician status was associated with appreciation, whereas autonomy did not consistently improve music evaluation.	Focused on characteristics attributed to the AI composer rather than direct comparison of AI and human musical sources.
Agudo et al. (2022)	Experiment 1: *N* = 249; Experiment 2: *N* = 250	Two between-subjects experiments using audiovisual artwork and a purely musical work	Identical material described as created by either AI or a human	Perceived emotion, creator sensitivity, quality and acceptance of AI creativity	AI attribution reduced perceived creator sensitivity and quality; in the purely musical experiment, the difference in the single-item emotion rating was not significant.	Each experiment used a limited set of artwork, and emotion was assessed with a relatively narrow measure.
Chu et al. (2022)	100 recruited; 83 valid respondents	Listening-based user evaluation of outputs from AI music-generation systems	Comparison among AI-generated outputs rather than AI-vs.-human authorship labels	Overall satisfaction, creativity, naturalness, melodiousness, richness, rhythm, correctness, structure and coherence	Listener ratings varied substantially across musical dimensions and generation systems; melodiousness and naturalness were important components of perceived quality.	Did not include a human-composed comparison or isolate authorship-attribution effects.
Shank et al. (2023)	Five experiments; final samples: *N* = 295, 399, 136, 393 and 136	Attribution-judgment and source-label experiments using electronic and classical excerpts	Human-composed music was judged or labeled as AI- vs. human-composed	Liking, perceived quality, valence, arousal, effort and perceived authorship	AI-composer bias was more apparent for classical or human-sounding music and was weak or absent for some electronic music conditions; valence and arousal were less consistently affected than liking and quality.	Experimental music was human-composed, so the findings primarily concern attribution bias rather than the quality of actual AI-generated music.
Yin et al. (2023)	*N* = 50 musically knowledgeable listeners	Blind comparison of human and algorithmic classical string quartets and piano improvisations	Actual algorithmically generated vs. human-composed excerpts	Aesthetic pleasure, stylistic success, repetition, melody, harmony and rhythm	Human-composed excerpts generally received stronger evaluations; deep-learning systems did not consistently outperform earlier algorithmic approaches.	Restricted to classical-style tasks and musically knowledgeable listeners; differences could reflect system and production quality.
Sarmento et al. (2024)	*N* = 32 progressive-metal listeners	Mixed-method listening study using progressive metal and rock excerpts	AI- vs. human-composed material, with genre and curation conditions	Preference, creativity, consistency, playability, perceived humanness and willingness to listen again	Some selected AI excerpts approached human-composed music on particular dimensions, but listeners generally preferred human compositions.	Small and highly genre-specific fan sample limits generalizability.
Ansani et al. (2025)	*N* = 120	Within-subject audiovisual crossover study using classical piano performances with identical audio	The same audio was framed as performed by a human pianist or an autonomous AI piano	Liking, quality, valence, arousal, engagement and perceived emotionality	The identical audio received lower liking, quality, engagement and emotional evaluations when attributed to AI; arousal showed little or no difference.	Findings are limited to classical piano and may partly reflect the audiovisual presentation of the performer.
Chia et al. (2025)	Final *N* = 64	Within-subject experiment using eight AI-generated pop songs	The same AI-generated songs were randomly labeled as AI- or human-generated	Liking, quality, positive emotion, sensorial and imaginal response, willingness to re-experience and purchase	No general negative AI-label effect was found; AI-labeled songs received higher ratings on positive emotion, while most other evaluations did not differ.	Small sample, one popular-music genre and one generation platform limit generalizability.
Dunham et al. (2025)	Study 1: *N* = 50; Study 2: *N* = 372	Two lab-in-the-field attribution experiments using the same musical material	Music was described as having an AI or human origin	Appreciation, emotional intensity, parasympathetic activity, perceived threat and beliefs about creativity	AI-origin beliefs reduced appreciation and emotional intensity and were associated with reduced parasympathetic activity; effects were stronger among listeners who regarded creativity as uniquely human.	The physiological pattern was interpreted as threat-related but may not generalize beyond the specific stimuli, setting and listener beliefs.
Fišer et al. (2025)	*N* = 88	Within-subject audiovisual experiment comparing three soundtrack conditions	Human-composed soundtrack vs. AI keyword-prompt and AI dimensional-prompt soundtracks	Pupil dilation, blink rate, skin impedance, valence, arousal, familiarity and audiovisual congruence	AI soundtracks produced greater pupil dilation and, in some conditions, higher arousal or biometric activation; valence did not differ significantly, and human music was more familiar.	Different musical materials were used across conditions, confounding AI origin with prompt, composition and production quality.
Gao et al. (2025)	*N* = 22	Evaluation of 75 clips generated by MusicLM, Stable Audio and MusicGen	Comparison of AI models, generation modalities and four intended emotion categories	Emotion-conveyance accuracy and induced emotion	“Energetic” was conveyed more accurately than several low-arousal categories; model performance varied, and recognized emotion was associated with felt emotion.	Very small sample, only four emotion categories and model-version-specific stimuli.
Rigole et al. (2025)	*N* = 57	Survey and listening-identification study using human, AI-co-created and fully AI-generated tracks	Comparison of human, co-created and AI-generated origins	Source identification, confidence, authenticity, emotional depth, labeling attitudes and willingness to engage	Co-created tracks were particularly difficult to identify; participants generally supported disclosure and showed cautious openness to human–AI collaboration.	Convenience sample, predominantly descriptive analyses and limited control over stimulus comparability.
Edelblum and Poe (2026)	Multiple experiments; pilot *N* = 194 and Study 1 *N* = 367	Consumer-listening experiments involving disclosure and direct exposure to AI-generated songs	AI-vs.-human source information presented before or after musical exposure	Expected enjoyment, perceived quality, willingness to replay, explore or share, and evaluative conflict	Initial stated resistance to AI music weakened after direct listening, although some reluctance to endorse or behaviorally support AI music remained.	Consumer-marketing context and heterogeneous experimental conditions complicate direct comparison with emotion-induction studies.
Wu and Holmes (2026)	Study 1: *N* = 99; Study 2: *N* = 300	Two preregistered studies using instrumental human-composed and AIVA-generated pieces	Correlational composer beliefs and a 2 × 2 manipulation of actual composer and human-vs.-AI label	Narrative listening, narrative engagement, perceived communicative intention and general impressions	AI attribution reduced the number of imagined narratives and narrative engagement, regardless of the music's actual composer.	Short instrumental clips and an online US sample limit conclusions about long-term personal meaning or attachment.
Chen and Collins (2026)	117 participants recruited; 101 included in the final analysis	Experimental comparison of Japanese pop excerpts in vocal and instrumental forms	AI-generated vs. human-composed music crossed with vocal vs. instrumental presentation	Emotion perception, emotional intensity and authorship identification	Participants had difficulty distinguishing AI from human music, but human-composed excerpts were judged as more emotionally intense.	Different audio was used across source conditions, and the findings may be specific to Japanese pop and vocal-content conditions.
Qi et al. (2026)	300 recruited; 283 valid responses	Comparative emotion-recognition study using 16 human-composed and 16 Suno-generated instrumental pieces	Actual AI-generated vs. human-composed music targeting four discrete emotions	Recognition accuracy for ecstasy, rage, grief and terror	Human music produced higher recognition accuracy for ecstasy, rage and terror; no significant difference was found for grief.	Single educational sample, forced-choice emotion categories and unmatched AI and human musical materials.

Identical-audio label studies isolate source-attribution effects more directly, whereas comparisons using different AI-generated and human-created stimuli may also reflect differences in composition, performance, familiarity, model version, or production quality. AI = artificial intelligence.

## 3 Conceptual definitions and dual-process framework

### 3.1 Forms of AI involvement in music

AI involvement in music can take several conceptually distinct forms, and these forms should not be treated as interchangeable.

The first is *fully automated generation*, in which an AI system produces a complete or nearly complete musical output from textual, melodic, stylistic, or other computational prompts with limited direct human intervention during the generation stage. Emotion-conditioned generation systems, for example, can map target affective categories onto musical parameters such as rhythm, pitch, note density, and mode (Ning et al., 2024; Zheng et al., 2022).

The second form is *AI-assisted creation*, in which a human creator uses AI to generate, transform, harmonize, arrange, orchestrate, or refine selected elements of a musical work while retaining primary control over the artistic process. In this case, AI functions mainly as a compositional or production tool rather than as an autonomous creator.

The third form is *human–AI co-creation*, in which human and computational agents contribute iteratively to musical ideation, generation, selection, revision, and arrangement. Human–AI co-creation usually involves repeated human aesthetic judgment rather than a single automated generation step. Research on AI-assisted songwriting competitions indicates that human participants frequently select, reject, modify, and reorganize model-generated materials during the creative process (Huang et al., 2020).

The fourth form is *AI performance*, in which the musical composition may be human-created, but its realization or performance is attributed to an autonomous or computational performer. AI-composer bias and AI-performer bias should therefore be distinguished because beliefs about composition and performance may activate different expectations concerning creativity, embodiment, expressiveness, and technical skill (Ansani et al., 2025; Shank et al., 2023).

The fifth form is *AI attribution or labeling*. In attribution studies, the musical material does not necessarily differ acoustically across experimental conditions. Instead, the same music may be described as AI-generated, AI-performed, human-composed, or human-performed. Such designs examine the psychological consequences of source beliefs rather than the objective properties of AI-generated music. Existing identical-audio studies show that source labels can influence liking, perceived quality, emotional intensity, engagement, or meaning-making even when the acoustic stimulus remains unchanged (Ansani et al., 2025; Dunham et al., 2025; Shank et al., 2023).

These forms of AI involvement may overlap in practice. Nevertheless, distinguishing actual AI generation, AI assistance, co-creation, AI performance, and source attribution is necessary because each supports a different type of empirical inference.

### 3.2 Emotional response, emotional alignment, emotional resonance, and aesthetic evaluation

Several related but non-equivalent constructs appear in research on AI-generated music. Clear differentiation among these constructs is necessary to avoid treating all favorable or unfavorable listener responses as evidence of emotional resonance.

In this review, *emotional response* refers to the listener's immediate affective, attentional, or physiological reaction to music. It includes felt valence, arousal, emotional intensity, attentional engagement, pupil dilation, electrodermal activity, heart-rate-related responses, and other autonomic indicators. Emotional responses to music can arise through multiple interacting mechanisms, including acoustic appraisal, expectancy, emotional contagion, memory, and physiological activation (Juslin and Västfjäll, 2008; Scherer, 2005).

*Emotional alignment* refers to the correspondence between an emotion encoded, intended, or experimentally targeted in a musical stimulus and the emotion recognized or experienced by the listener. This construct requires a distinction between *emotion conveyance or recognition* and *emotion induction*. Emotion conveyance concerns whether listeners correctly identify the emotion that the music appears to express. Emotion induction concerns whether listeners themselves experience that emotion. A listener may therefore recognize that a musical excerpt expresses sadness without personally feeling sad (Juslin and Västfjäll, 2008). Studies of AI-generated music should not treat successful recognition of an intended emotional category as equivalent to the induction of the same emotion in the listener (Gao et al., 2025).

*Aesthetic evaluation* refers to reflective judgments concerning the artistic or experiential value of music. Typical measures include liking, perceived quality, creativity, authenticity, expressiveness, coherence, naturalness, artistic value, and willingness to listen again. These evaluations may be influenced by structural properties of the music, source beliefs, listener expectations, familiarity, and assumptions about creative agency (Shank et al., 2023; Thompson et al., 2023). Aesthetic evaluation is related to emotional response but is not equivalent to it. A listener may experience strong arousal without liking the music, or may judge a work as technically impressive without experiencing strong emotion.

*Emotional resonance* refers to a broader and potentially deeper form of listener engagement involving personal meaning, autobiographical relevance, perceived understanding, narrative meaning-making, source-based empathy, sustained attachment, or significance developed through repeated listening. This definition is consistent with broader accounts of music appreciation that emphasize not only musical structure but also the listener's sense of self and beliefs about the source of the music (Thompson et al., 2023).

Most existing studies of AI-generated or AI-attributed music have measured immediate affective response, emotion recognition, or aesthetic evaluation rather than emotional resonance directly. Measures such as liking, arousal, authenticity, or perceived quality should therefore not be treated individually as complete indicators of emotional resonance. In this review, emotional resonance is used as an overarching theoretical construct whose deeper components remain insufficiently tested.

The review also distinguishes between *computationally represented emotion* and *human-experienced emotion*. AI systems can model statistical relationships between musical features and emotion labels, including associations between tempo, mode, pitch range, loudness, timbre, rhythmic density, and perceived valence or arousal (Gabrielsson and Lindström, 2010; Ning et al., 2024; Zheng et al., 2022). However, the generation of acoustically recognizable emotional cues does not imply that an AI system possesses subjective feeling, autobiographical experience, communicative intention, or emotional understanding. Statements such as “AI expresses sadness” therefore refer only to the generation of musical cues conventionally associated with sadness.

### 3.3 Dual-process framework

To organize the available evidence, this review proposes a dual-process framework comprising a relatively low-level acoustic–affective pathway and a higher-level source-based appraisal pathway. The two pathways are partially dissociable but not fully independent.

The *acoustic–affective pathway* begins with properties of the musical stimulus, including tempo, mode, timbre, loudness, pitch range, rhythmic density, harmonic tension, structural predictability, and production quality. These features can support relatively rapid emotion recognition, felt valence, arousal, attentional engagement, and autonomic response (Gabrielsson and Lindström, 2010; Juslin and Västfjäll, 2008). Studies of AI-generated music indicate that generated excerpts can convey some intended emotional categories and produce measurable affective, attentional, or physiological responses under specific conditions (Fišer et al., 2025; Gao et al., 2025).

The *source-based appraisal pathway* begins with information or beliefs concerning the identity of the composer, performer, or production system. An AI-authorship or AI-performance label may activate judgments about agency, intentionality, authenticity, effort, embodiment, creativity, and human experience. These appraisals can influence liking, perceived quality, expressiveness, emotional engagement, and emotional intensity even when the underlying audio remains unchanged (Ansani et al., 2025; Dunham et al., 2025; Shank et al., 2023).

The two pathways may interact. Source information can alter reported emotional intensity or engagement, while production quality or genre-incongruent musical features can influence higher-order evaluations that might otherwise be attributed to AI bias. Similarly, a listener may experience strong arousal through acoustic processing while simultaneously assigning low authenticity or artistic value through source-based appraisal. Divergence between these pathways may help explain why AI attribution sometimes reduces liking or perceived quality without consistently reducing valence, arousal, or physiological activation (Chia et al., 2025; Fišer et al., 2025).

The strength and direction of both pathways may be moderated by musical genre, cultural background, musical expertise, familiarity, attitudes toward AI, anthropomorphic tendencies, beliefs about whether creativity is uniquely human, and prior exposure to AI-generated music. However, many of these moderators remain insufficiently tested and should be treated as boundary conditions and future research questions rather than established effects.

**Figure 2:**
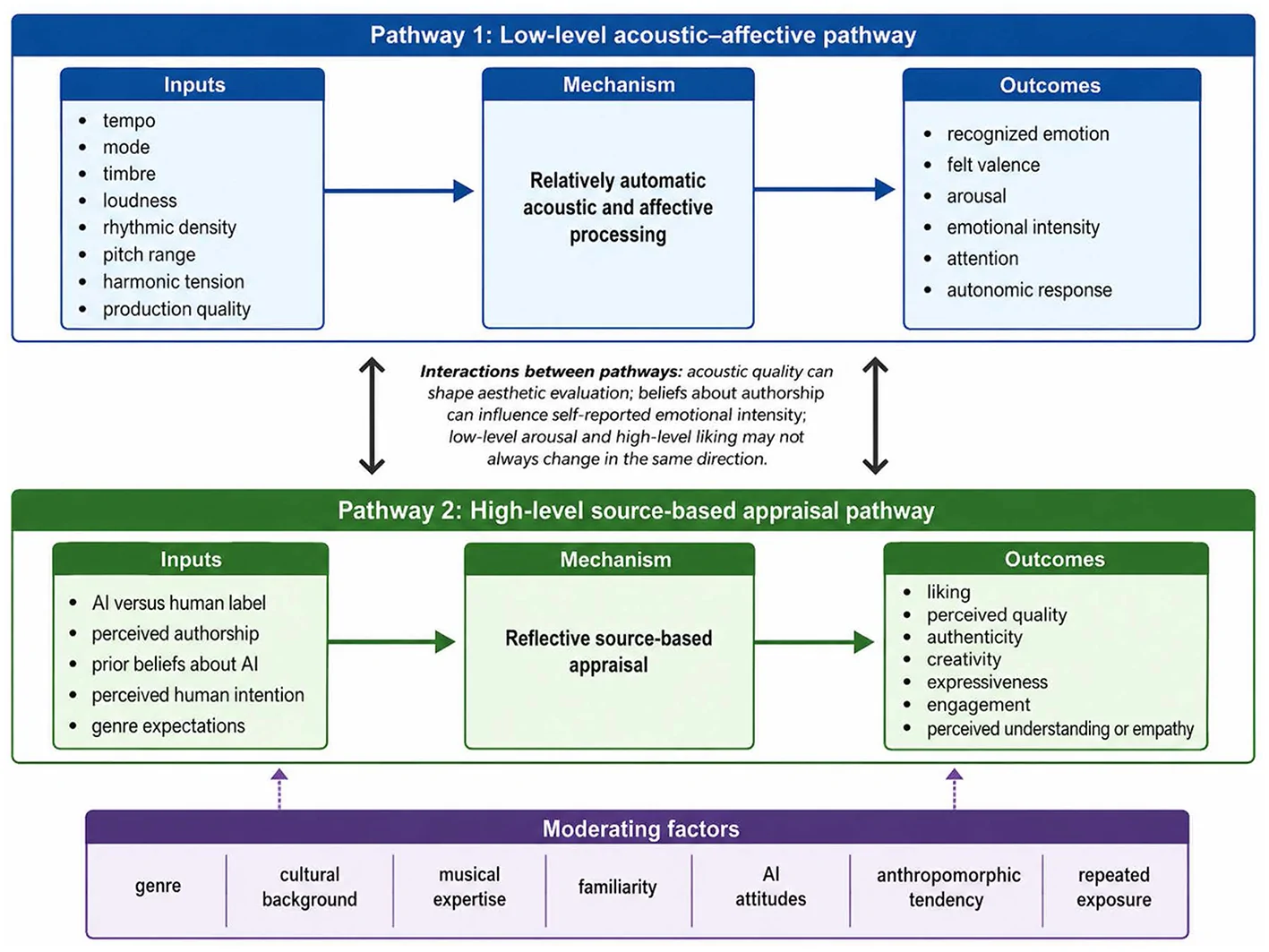
Dual-process framework of listener responses to AI-generated and AI-attributed music. *Note*. Solid arrows represent the proposed directional pathways from inputs through processing mechanisms to listener outcomes. Double-headed arrows indicate interactions between the two pathways, and dotted arrows indicate potential moderating effects.

Figure 2 summarizes the proposed relationships among acoustic features, source attribution, listener appraisal, affective responses, aesthetic evaluation, and potential moderating factors.

## 4 Empirical evidence synthesis

### 4.1 Authorship attribution and source-label effects

Authorship-attribution studies examine whether listeners respond differently to music when they believe that it was created or performed by AI rather than by a human. The clearest evidence comes from identical-audio designs, in which the acoustic stimulus is held constant and only the purported source is manipulated. Such designs permit stronger inferences about source-label and expectancy effects than comparisons involving different AI-generated and human-created excerpts.

Shank et al. (2023) found that the relationship between perceived AI authorship and music evaluation depended on musical genre and the extent to which the excerpts conformed to listeners' expectations of AI-generated music. In their electronic-music condition, experimentally assigning an AI rather than a human composer did not consistently reduce liking. By contrast, when human-sounding classical excerpts were presented as AI-composed, participants reported lower liking than when the same excerpts were presented as human-composed. These results suggest that AI-composer bias is conditional rather than uniform and may be stronger when an AI label conflicts with genre-specific expectations concerning human creativity and expressiveness.

A similar pattern has been reported for musical performance. Ansani et al. (2025) used a crossover audiovisual design in which participants evaluated three classical piano performances presented in human-performer and autonomous-AI-performance conditions, while the audio remained identical. Performances attributed to the human pianist were rated as more likable, more engaging, higher in emotional valence, and higher in perceived quality. Arousal did not show the same clear source-label difference. The findings therefore indicate that beliefs about the performer can shape aesthetic and emotional evaluation even when listeners hear identical musical material.

Dunham et al. (2025) similarly examined responses to the same music described as having either an AI or a human origin. AI-origin beliefs were associated with lower appreciation and lower reported emotional intensity, together with changes in parasympathetic activity. The authors interpreted the physiological pattern as being consistent with heightened threat-related responding. However, this interpretation derives from a specific attribution paradigm and should not be generalized to all AI-generated music, genres, or listener populations.

Evidence is not uniformly negative. Chia et al. (2025) found no general devaluation of AI-labeled pop music and reported higher positive-emotion ratings for some AI-labeled excerpts. These findings indicate that source-label effects may vary according to genre, listener attitudes, prior expectations, stimulus quality, and the particular outcome being measured. Taken together, the available studies suggest that AI attribution can influence higher-order evaluations such as liking, quality, engagement, authenticity, and meaning-making, but the direction and magnitude of the effect are not consistent across all listening contexts.

Other attribution-related studies further indicate that source information can influence creator sensitivity, narrative engagement, expected enjoyment, and acceptance of AI as a legitimate musician, although these effects vary across outcomes and experimental contexts (Agudo et al., 2022; Edelblum and Poe, 2026; Hong et al., 2022; Tigre Moura and Maw, 2021; Wu and Holmes, 2026).

### 4.2 Emotional conveyance, induction, and direct stimulus comparisons

Unlike source-label studies, the studies reviewed in this section generally compare actual AI-generated outputs with human-created music or compare outputs from different AI systems. These designs can assess the emotional or aesthetic effectiveness of generated material, but they cannot isolate source-attribution effects because the musical stimuli differ across conditions.

The simulation of basic emotional cues by artificial intelligence mainly relies on the established associations between acoustic features and perceived emotion in psychoacoustics. Research shows that features such as tempo, mode, loudness, pitch range, rhythmic density, and timbral brightness are associated with listeners' judgments of musical emotion (Gabrielsson and Lindström, 2010; Juslin and Västfjäll, 2008). In AI music generation, developers translate these relationships into computationally controllable parameters. For example, EmotionBox maps happiness, tension, sadness and calmness into a two-dimensional valence–arousal space, and conditions the generated output toward target emotion categories through musical parameters such as mode and note density (Zheng et al., 2022). Similarly, Ning et al. (2024) generated emotion-conditioned music by separating rhythm and tone characteristics. This shows that the emotional simulation of artificial intelligence comes from statistical modeling between acoustic features and emotional labels, rather than subjective emotional understanding.

Gao et al. (2025) compared emotional conveyance and induction across outputs generated by MusicLM, Stable Audio, and MusicGen. Within the models and emotion categories examined, “Energetic” was conveyed more accurately than several lower-arousal categories, including “Sluggish” and “Peaceful,” while model performance also varied. These findings should not be generalized to all generative systems, musical genres, or emotion taxonomies. However, evidence that current systems can consistently convey complex or mixed emotions remains limited. Cowen et al. (2020) identified at least 13 dimensions of subjective musical experience, extending beyond simple happiness–sadness or valence–arousal distinctions. Emotions such as nostalgia, awe or dreaminess largely depend on cultural memories, personal experiences and musical expectations. Evidence concerning complex, mixed, culturally embedded, or autobiographically meaningful emotions remains limited. The available studies primarily assess broad categories or dimensions such as happiness, sadness, valence, arousal, energy, and peacefulness.

Fišer et al. (2025) extended this evidence to an audiovisual context by comparing human-composed soundtracks with soundtracks generated under two AI-prompting conditions. AI-generated soundtracks were associated with greater pupil dilation and, in some conditions, higher self-reported arousal, whereas valence did not differ significantly across conditions. These findings indicate that AI-generated music can elicit measurable attentional and physiological activation. However, because the human and AI conditions used different musical materials, the observed differences cannot be attributed solely to authorship or source beliefs. They may also reflect variation in composition, prompt quality, audiovisual congruence, familiarity, production quality, or model performance.

Direct comparisons across classical, popular, progressive-metal, and instrumental materials similarly show that AI-generated music can convey recognizable emotions and sometimes approach human-created music on selected dimensions, but human-created music often retains advantages in emotional intensity, stylistic success, or overall preference (Chen and Collins, 2026; Qi et al., 2026; Sarmento et al., 2024; Yin et al., 2023). Other studies emphasize substantial variation across generation systems and the difficulty of reliably identifying human, AI-generated, and co-created music (Chu et al., 2022; Rigole et al., 2025).

### 4.3 Potential moderators and individual-difference boundary conditions

Individual differences have been proposed as potential moderators of responses to AI-generated and AI-attributed music, but relatively few studies have tested these moderators systematically. Musical expertise, familiarity with digital music production, attitudes toward AI, anthropomorphic tendencies, and beliefs about whether creativity is uniquely human should therefore be treated as potential boundary conditions rather than as established determinants.

Musical expertise may influence the criteria used to evaluate AI-generated music. Less-experienced listeners may rely more strongly on familiarity, immediate enjoyment, or general emotional impression, whereas trained musicians may attend more closely to structural coherence, harmonic development, timbral control, expressive timing, and performance nuance. However, the current evidence does not establish that expertise consistently strengthens or weakens AI-authorship bias. Indeed, Ansani et al. (2025) reported that the source-label effects in their performance study were not significantly moderated by musical expertise.

Attitudes toward technology and anthropomorphic tendencies may also influence acceptance, although these moderators have not been tested consistently across studies. Listeners who are familiar with digital production or who regard AI as a legitimate creative tool may show weaker negative source-label effects. Conversely, listeners who believe that musical creativity necessarily depends on human consciousness, embodiment, or lived experience may evaluate AI-attributed music less favorably. In Ansani et al. (2025), responses were moderated by participants' attitudes toward AI, while Dunham et al. (2025) suggested that beliefs about the uniquely human nature of creativity may be relevant to negative responses to AI-origin information. These findings are suggestive but do not justify treating AI attitudes or anthropomorphic tendencies as universal moderators.

Other variables, including age, cultural background, genre familiarity, prior exposure to AI-generated music, and listening motivation, remain insufficiently examined. Future studies should preregister moderation hypotheses, use adequately powered samples, and distinguish exploratory individual-difference associations from replicated moderator effects.

## 5 Critical synthesis and methodological limitations

Overall, the available evidence suggests that AI-authorship attribution can influence liking, perceived quality, authenticity, engagement, emotional intensity, and meaning-making. However, effects on valence, arousal, emotion induction, and physiological activity are less consistent. Current findings therefore support neither the claim that AI-generated music is incapable of meaningful emotional impact nor the claim that listeners respond to AI-generated and human-created music equivalently. Source information appears to influence reflective and aesthetic evaluations more consistently than immediate affective or physiological responses.

### 5.1 Limited ecological and temporal validity

Most studies examine immediate responses to short musical excerpts presented in laboratory or online settings. Although such designs can isolate short-term attribution effects, they do not examine how emotional significance develops through repeated listening, autobiographical association, social context, or familiarity. Consequently, current evidence concerning sustained attachment, personal meaning, perceived understanding, and long-term emotional resonance remains limited.

### 5.2 Acoustic and production-quality confounds

Direct comparisons of AI-generated and human-created music may be confounded by differences in composition, mixing, loudness, timbre, structural coherence, expressive timing, familiarity, and production quality. For example, the conditions examined by Fišer et al. (2025) differed not only in musical origin but also in prompt, composition, audiovisual congruence, and familiarity. Lower ratings of AI-generated music therefore cannot always be attributed to AI authorship itself. Future studies should match or statistically control relevant acoustic and production characteristics.

### 5.3 Source attribution vs. actual stimulus differences

Identical-audio label studies and direct AI-vs.-human comparisons support different inferences. When the same audio is assigned different source labels, differences can be interpreted more confidently as source-label or expectancy effects (Ansani et al., 2025; Dunham et al., 2025; Shank et al., 2023). When different AI-generated and human-created excerpts are compared, the results may also reflect differences in musical quality, model version, or genre fit. Future factorial designs should manipulate both actual source and attributed source.

### 5.4 Construct and measurement limitations

The reviewed outcomes are related but not interchangeable. Liking is not equivalent to emotional resonance; arousal does not necessarily indicate engagement; authenticity does not directly measure empathy; and perceived quality is not a measure of felt emotion. Emotion recognition should also be distinguished from emotion induction because recognizing that music expresses sadness does not mean that the listener feels sad (Gao et al., 2025; Juslin and Västfjäll, 2008). Physiological measures require caution: pupil dilation may indicate attention, novelty, arousal, or cognitive effort rather than positive or negative emotion. Moreover, few studies directly measure attachment, autobiographical meaning, perceived understanding, or source-based empathy.

### 5.5 Genre and cultural boundary conditions

Genre may shape responses to AI authorship. Shank et al. (2023) reported clearer negative label effects for classical than for electronic music. Classical music may evoke stronger expectations concerning human intentionality, virtuosity, and expressive agency, whereas electronic music is already associated with synthesis and digital production. This interpretation remains provisional because the proposed mechanisms were not measured directly. Cultural learning may also shape musical emotion, authenticity judgments, and beliefs about technological creativity. Because many studies use university students or online convenience samples from limited cultural settings, their findings should not be generalized automatically. Cross-genre and cross-cultural replication is therefore needed.

## 6 Future agenda and conclusion

Based on the current evidence, future research should address four priorities. First, future research should move from single-exposure studies using short excerpts to longitudinal and repeated-listening designs. Researchers should examine whether listeners develop sustained emotional attachment, familiarity-based preferences, or parasocial relationships with perceived creators through repeated exposure to AI-generated music. Current research explains how artificial intelligence labels affect immediate preferences and quality judgment, but current evidence does not allow firm conclusions about whether long-term emotional resonance is enhanced or weakened (Ansani et al., 2025; Shank et al., 2023).

Second, future experiments must strictly distinguish between the authorship-label effect and the audio quality effect. The strongest design should use the same audio but different source labels, or strictly match the volume, mixing, style, familiarity and production quality of AI-generated music and human music. Otherwise, MIDI-like timbre or poor generation quality may lead to low scores, rather than genuine AI-related bias.

Third, the research should systematically incorporate the moderators of individual differences. These factors include musical expertise, familiarity with digital production, attitudes toward AI, anthropomorphic tendencies, beliefs about whether creativity is uniquely human, and prior exposure to AI-generated music. Preliminary evidence suggests that beliefs about whether creativity is uniquely human may shape responses to AI-origin information. However, these moderators require direct and adequately powered tests rather than reliance on overall mean differences. Therefore, simply reporting the overall average difference will obscure meaningful individual differences (Dunham et al., 2025).

Fourth, cross-genre and cross-cultural replications should examine whether AI-authorship effects vary according to genre expectations, cultural background, musical training, familiarity, and production quality.

Returning to the dual-process framework, current evidence suggests two partially dissociable but interacting pathways. Through the acoustic–affective pathway, musical features such as tempo, mode, loudness, timbre, rhythmic density, and harmonic tension may shape emotion recognition, valence, arousal, attention, and autonomic responses. Through the source-based appraisal pathway, beliefs about AI authorship may influence perceived agency, intentionality, authenticity, effort, emotional intensity, and perceived understanding. These effects are not uniformly negative and may vary across genres, outcomes, and listener characteristics.

Therefore, the basic conclusion of this article is that the music generated by artificial intelligence can simulate several acoustic cues related to basic emotional categories, and can trigger measurable emotions, attention or physiological reactions in specific tasks and audio-visual situations. However, the current evidence more clearly supports differences in higher-level cognitive assessment than differences in basic arousal or valence responses. Whether the music generated by artificial intelligence can support deeper emotional resonance, such as attachment, perceived understanding, personal meaning or autobiographical relevance or source-based empathy, has not been fully confirmed. The key question of future research is no longer just “Does AI-generated music have emotions?” Rather, researchers should examine the task conditions, musical genres, model quality, listening histories, and listener characteristics under which AI-generated music supports emotion recognition, emotion induction, and potentially deeper emotional resonance.
